# *Propionibacterium acnes *infection induces upregulation of inflammatory genes and cytokine secretion in prostate epithelial cells

**DOI:** 10.1186/1471-2180-10-126

**Published:** 2010-04-26

**Authors:** Johanna B Drott, Oleg Alexeyev, Patrik Bergström, Fredrik Elgh, Jan Olsson

**Affiliations:** 1 Department of Clinical Microbiology/Virology, Umeå University, SE-901 87 Umeå, Sweden; 2 Department of Clinical Medicine, School of Health and Medical Sciences, Örebro University, SE-701 82 Örebro, Sweden

## Abstract

**Background:**

The immune stimulating bacterium Propionibacterium acnes is a frequent colonizer of benign and malignant prostate tissue. To understand the pathogenesis of the earliest phase of this infection, we examined the P. acnes triggered immune response in cultivated prostate epithelial cells.

**Results:**

Prostate epithelial cells are triggered to secrete IL-6, IL-8 and GM-CSF when infected with P. acnes. The secretion of cytokines is accompanied by NFκB related upregulation of the secreted cytokines as well as several components of the TLR2-NFκB signaling pathway.

**Conclusions:**

*P. acnes *has potential to trigger a strong immune reaction in the prostate glandular epithelium. Upon infection of prostate via the retrograde urethral route, the induced inflammatory reaction might facilitate bacterial colonization deeper in the prostate tissue where persistent inflammation may impact the development of prostate diseases as hyperplasia and/or malignancy.

## Background

Asymptomatic histological inflammation is a common feature when prostate tissue is subjected to morphological examination. Varying degree of inflammation is present at both benign (prostatic hyperplasia) and malignant (neoplasia) conditions. A growing amount of research supports the idea that chronic prostatic inflammation contributes to gradual transition of normal epithelial cells to malignant cells [1]. For example, many of the gene-variants linked to familiar prostate cancer code for proinflammatory cytokines and chemokines [2]. A plethora of microorganisms have been evaluated for their possible involvement in the etiology of prostate inflammation. Many studies purported *E. coli *and sexually transmitted agents as likely candidates capable of inducing chronic prostatic inflammation [3-5]. A Gram-positive bacterium; *Propionibacterium acnes *(*P. acnes*) has been reported to be frequently present in various prostatic diseases (as reviewed in [6]) and its presence has been correlated to inflammation in prostate cancer specimens [7-9]. *P. acnes*, a well studied pathogenetic factor in cutaneous disorders like acne vulgaris, has been demonstrated to stimulate monocytes and endothelial cells to secrete pro-inflammatory cytokines via activation of Toll-like receptor (TLR) 2 [10,11]. In this study we present an *in vitro *model to study the inflammatory response of prostate derived epithelial cells to *P. acnes *infection. We report that *P. acnes *induces upregulation of numerous pro-inflammatory substances at the mRNA level accompanied by secretion of respective soluble substances such as interleukins 6, 8 and GM-CSF. Components of the TLR2-NFκB signaling pathway were upregulated, suggesting an involvement of this particular pathway for the response. Blocking of the TLR2 with monoclonal antibodies partly reduced the effects.

## Results

### Pilot studies to define experimental conditions for *P. acnes *infection of epithelial cells

Secretion of cytokines is one of the end results of innate immune response at a cellular level. We therefore assessed the secretion of three key cytokines, IL-6, IL-8 and GM-CSF (also called CSF-2) from the prostate-derived epithelial cell-line RWPE-1 in response to infection with *P. acnes*. To set experimental conditions as multiplicity of infection (MOI) and useful infection time, we defined the desired criteria as maximal cytokine secretion after 48 h and no visual cellular detachment or cell-death. A MOI of 16-40:1 fulfilled these criteria (data not shown). We therefore decided to use a MOI of 16:1 for the following experiments.

### Prostate epithelial cells secrete IL-6, IL-8, and GM-CSF in response to *Propionibacterium acnes *infection

**Figure 1:**
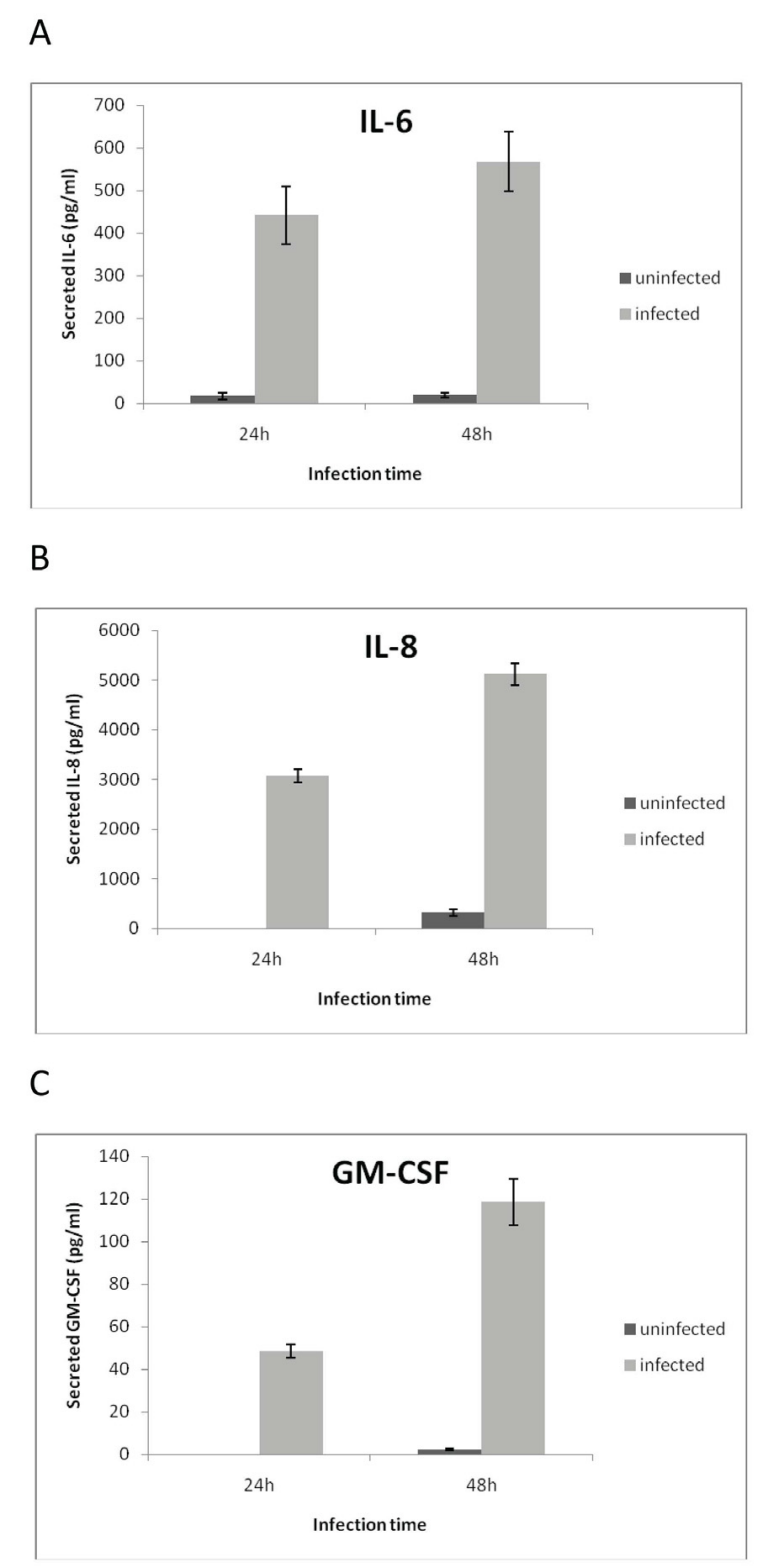
***P. acnes*-induced secretion of IL-6 (a), IL-8 (b) and GM-CSF (c) by RWPE-1 cells at 24 h and 48 h after infection**. Semiconfluent RWPE-1 monocell-layers were infected with *P. acnes *at a MOI of 16:1. Cytokines released into supernatants were quantified by ELISA. The diagram shows means for triplicates with the error bars representing the standard deviation.

Secretion of cytokines IL-6, IL-8 and GM-CSF by RWPE-1 was measured 24 h and 48 h after infection with *P. acnes*. 24 h after infection, the levels of secreted IL-6, IL-8 and GM-CSF were: 441.7 ± 67.6, 3071.1 ± 133.7, and 48.6 ± 3.1 (pg/ml), respectively. The corresponding values from the uninfected control cells were: 17.0 ± 8.0 (pg/ml), not detectable, not detectable (Figure 1). 48 h after infection, the concentrations increased to: 567.7 ± 70.7, 5121.5 ± 218.0, and 118.6 ± 10.6 (pg/ml). Uninfected: 19.9 ± 5.8, 320.6 ± 71.4, and 2.1 ± 0.5 (pg/ml). The diagram shows means for triplicates with the error bars representing the standard deviation [12] (Figure 1).

### *P. acnes *induced secretion of IL-8 is partially blocked by α-TLR-2 antibodies

**Figure 2:**
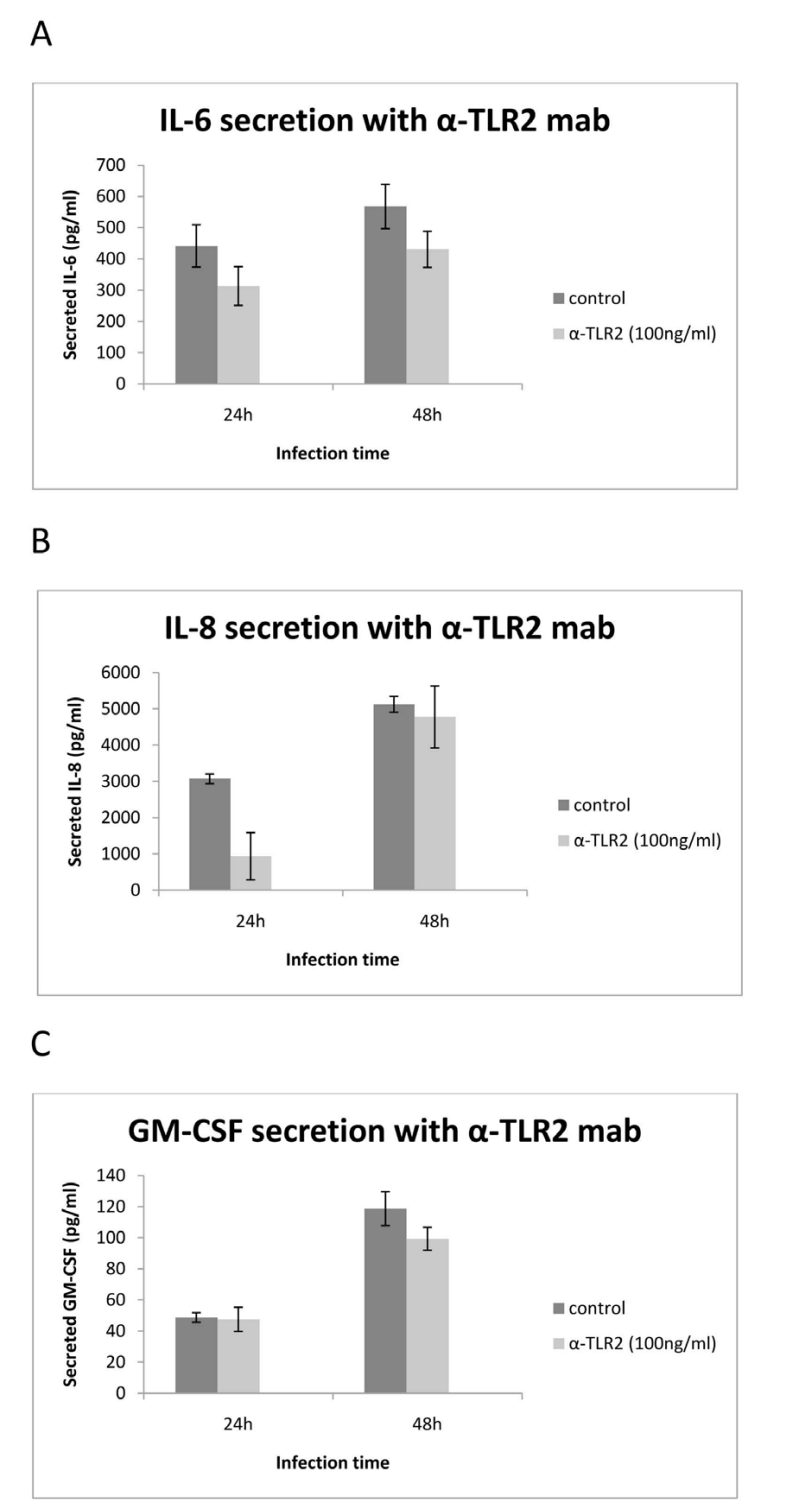
**α-TLR2 inhibition of IL6, IL-8 and GM-CSF secretion by *P. acnes*-infected RWPE-1**. α-TLR2 mouse monoclonal antibodies (100 ng/ml) were added one hour prior to *P. acnes *infection of semiconfluent RWPE-1 monocell-layers. Supernatants were collected at 24 h and 48 h after infection. The amount of cytokines released into the medium was quantified by ELISA. The diagram shows means for triplicates with the error bars representing the standard deviation.

To determine whether the secretion of IL-6, IL-8, and GM-CSF was TLR2-mediated, TLR2 on RWPE-1 cells were blocked with monoclonal anti-TLR2 antibodies at a concentration of 100 ng/ml prior to infection. This particular mab clone has previously been demonstrated to block TLR2 activation in human cells [13]. Secretion of IL-8 was significantly (*p *= 0.05) reduced when measured 24 h after infection (Figure 2). No such blocking effect was recognizable 48 h after infection. Levels of IL-6 and GM-CSF were not significantly affected (Figure 2). Figure 2 shows means for triplicates with the error bars representing the standard deviation.

### *P. acnes *infection induces up-regulation of several cytokines and components of the TLR-2 signaling pathway

The potent *P. acnes *stimulated effect on secretion of IL-6, IL-8 and GM-CSF prompted us to investigate an array of genes involved in inflammatory signaling pathways. As our main focus is the early responses, we wanted to collect mRNA as early as possible, yet late enough to allow observation of significant regulatory events. We used the cDNA prepared from cells infected for 24 h for comparison with cDNA from uninfected cells. Of the 84 genes analyzed, 20 were more than two-fold upregulated (*p *= 0.05): CCL2, CSF2 (GM-CSF), CSF3, CXCL10, IFNB1, IL1A, IL6, IL8, IRAK2, IRF1, JUN, LTA, NFKB2, NFKBIA, REL, RELA, RIPK2, TLR2, TNF, and TICAM1 (Table 1). Only four genes were downregulated (p = 0.05): FOS, HMGB1, TLR4 and UBE2V1 (Table 2).

**Table 1:** List of genes that are upregulated upon *P. acnes *infection.

Gene name	Description	Fold upregulation
CCL2	Chemokine (C-C motif) ligand 2	41
CSF2	Colony stimulating factor 2 (granulocyte-macrophage)	139
CSF3	Colony stimulating factor 3 (granulocyte)	39
CXCL10	Chemokine (C-X-C motif) ligand 10	107
IFNB1	Interferon, beta 1, fibroblast	12
IL1A	Interleukin 1, alpha	12
IL6	Interleukin 6 (interferon, beta 2)	34
IL8	Interleukin 8	336
IRAK2	Interleukin-1 receptor-associated kinase 2	11
IRF1	Interferon regulatory factor 1	12
JUN	Jun oncogene	10
LTA	Lymphotoxin alpha (TNF superfamily, member 1)	5
NFKB2	Nuclear factor of kappa light polypeptide gene enhancer in B-cells 2 (p49/p100)	8
NFKBIA	Nuclear factor of kappa light polypeptide gene enhancer in B-cells inhibitor, alpha	6
REL	V-rel reticuloendotheliosis viral oncogene homolog	4
RELA	V-rel reticuloendotheliosis viral oncogene homolog A,	2
RIPK2	Receptor-interacting serine-threonine kinase 2	4
TLR2	Toll-like receptor 2	3
TNF	Tumor necrosis factor (TNF superfamily, member 2)	53
TICAM1	Toll-like receptor adaptor molecule 1	3

Semiconfluent RWPE-1 monocell-layers were infected with *P. acnes *at a MOI of 16:1. After 24 h infection, the cells were harvested, mRNA was collected and cDNA was prepared. The cDNA corresponding to 84 inflammation-associated genes was quantified with qRT-PCR and compared with cDNA prepared from non-infected cells. Inclusion criteria: > 2-fold up-regulation, (*p *= 0.05).

**Table 2:** List of genes that are downregulated upon *P. acnes *infection.

Gene name	Description	Fold upregulation
FOS	V-fos FBJ murine osteosarcoma viral oncogene homolog	-3
HMGB1	High-mobility group box 1	-3
TLR4	Toll-like receptor 4	-4
UBE2V1	Ubiquitin-conjugating enzyme E2 variant 1	-3

Semiconfluent RWPE-1 monocell-layers were infected with *P. acnes *at a MOI of 16:1. After 24 h infection, the cells were harvested, mRNA was collected and cDNA was prepared. The cDNA corresponding to 84 inflammation-associated genes was quantified with qRT-PCR and compared with cDNA prepared from non-infected cells. Inclusion criteria: > 2-fold down-regulation, (*p *= 0.05).

## Discussion

Prostate specimens commonly display signs of chronic histological inflammation, along with occasional acute inflammation. Numerous studies have explored a possible link between prostate inflammation and cancer development and recent reviews of epidemiologic, genetic, and molecular studies have collectively suggested that the two cellular processes may indeed interact [2,14-16]. Exposure to environmental factors such as infectious agents can lead to injury of the prostate and to the development of chronic inflammation [17]. The intrinsic interplay between microbes and urogenital cells is a key feature in the understanding of the microbial involvement in prostate disease. Both *Chlamydia *and *Mycoplasma *have been demonstrated to induce IL-6 and IL-8 production in immortalized normal prostate epithelial cells (PNT2) [18,19]. Given the emergence of *P. acnes *as an infecting agent in prostate tissue [7-9] we investigated the effect of the bacterium on prostate epithelial cells of non-malignant origin (RWPE-1). In vitro, *P. acnes *induced considerable secretion of IL-6 and IL-8 and, to a lesser extent, GM-CSF. Secretion of IL8 was shown to be mediated via TLR2, as the receptor blockage with anti-TLR2 monoclonal antibodies reduced its secretion. In contrast, we did not observe any significant reduction in secretion of IL-6 and GM-CSF by blockage of TLR2. Earlier reports present evidence that *P. acnes *is able to stimulate monocytes and endothelial cells to secrete pro-inflammatory cytokines via activation of TLR2 [10,11]. Our results partly confirm this. Even toll-like receptors 4 and 9 have been implicated in *P. acnes *mediated immune modulatory effects [20]. Both human and rat prostate epithelial cell lines are known to express TLR2, TLR4, and TLR9 [21,22] and since blockage of TLR2 in our experiment has not totally inhibited cytokine secretion, the involvement of other TLR may also be hypothesized. However, possible TLR4 involvement is compromised by the observed downregulation of the gene expression. Another mechanism may involve auto inducing capability of the released cytokines that generates a self-perpetuating inflammatory process.

The increased secretion of such cytokines was accompanied by concordant mRNA up-regulation. Moreover, the broader analysis of inflammation associated genes revealed that chemokine ligands and pro-inflammatory substances CCL2, CXCL10, TNF-α, TNF-β (lymphotoxin-α), CSF3, IL1-α, and IFN-β were also significantly upregulated. Further studies are required to determine if upregulation of aforementioned genes is accompanied by enhanced cytokine production by prostate epithelial cells. The upregulation of the transcriptional regulators JUN, REL, RIPK2, NFKB2, NFKBIA, IRF1, IRAK2 and the TLR/IL1-receptor co-factor TICAM1 is coherent with earlier studies of TLR2 signaling cascade leading to Fib activation [23,24].

Secretion of IL-6, IL-8 and GM-CSF are central for recruitment and differentiation of macrophages and neutrophils in inflamed tissue [25-27]. A prolonged time of increased cytokine levels might have adverse effects on the tissue. *P. acnes *induced elevation of IL-8 expression in hair-follicle endothelial cells is associated with epidermal hyperplasia and follicular hyperkeratosis in acne vulgaris and psoriasis [28,29]. There is also a correlation between the more pronounced IL-8 expression and dermal angiogenesis [29]. Interestingly, both IL-6 and IL-8 have been suggested as contributors to prostate cancer development. The expression of IL-6 and its receptor has been demonstrated in clinical specimens of both prostate cancer and benign prostate hyperplasia [30], and levels of IL-6 increase in organ-confined tumors [31]. *In vitro *experiments have shown that IL-6 may play a role in prostate cancer cell growth and differentiation and that it stimulates cell growth of malignant cells [32]. Following prolonged treatment with IL-6, prostate cancer cells can alter the responsiveness to the cytokine and acquire the ability to proliferate at a higher rate and become more tumorigenic [33,34]. IL-8 has been shown to increase the transcriptional activity of the androgen receptor in prostate cancer cell lines, suggesting a potential role of this chemokine in modulating the transition of prostate cancer to an androgen-independent state [35]. Other studies report that IL-8 contribution to prostate cell proliferation is independent of the androgen receptor [36]. Our data indicate that the prostate epithelium significantly contributes to locally increased levels of both IL-6 and IL-8 when infected with *P. acnes*, thus potentially promoting adverse effects as increased proliferation and angiogenic activities by autocrine and/or endocrine mechanisms. The pathogenesis of *P. acnes *in locations other than the hair-follicle is still poorly understood. We currently address questions about its involvement in prostate disease such as prevalence, genetic variability and impact on histological inflammation and neoplasia (Elgh *et al*., manuscripts in preparation).

## Conclusions

In conclusion, we demonstrate that prostate epithelial cells secrete inflammatory cytokines in response to *P. acnes*, partly through a TLR2-mediated mechanism. We propose that this strong immune-stimulating effect facilitates the bacterial colonization deeper into the prostate tissue where P. acnes can form long-lasting biofilm-like aggregates [7]. A possible mechanism may involve intracellular transport in recruited macrophages, as P. acnes has been demonstrated to withstand degradation by phagocytosing mononuclear cells [37].

## Methods

### Prostate cell lines

RWPE-1, human prostate epithelial cell line (ATCC^© ^CRL-11609) was maintained in complete KSF-medium supplemented with 5 ng/l EGF, 0.05 mg/l BPE and 100 U/ml PEST (GIBCO BRL/Life technologies, Inc., Gaithersburg, MD, USA).

Cells were split 1:5, 1-2 times per week using 0,05% (w/v) trypsin/EDTA (GIBCO BRL/Life technologies, Inc., Gaithersburg, MD, USA). Cells were maintained in a humidified incubator at 37C containing 5% CO_2_.

### *Propionibacterium acnes*

*P. acnes*, serotype 1a, isolated from craniopharyngeom fluid was grown in Brain-Heart Infusion Broth + 5% horse serum at 37C under microaerobic conditions. The bacteria were grown to a density of 10^9 ^per ml, pelleted and resuspended into sterile PBS.

### Cytokine ELISA

RWPE-1 cells were seeded into 24-well plates at a density of 1 × 10^5^cells per well in one ml normal growth medium. After 48 h, cells were washed in PBS and the medium was changed to DMEM without FCS and PEST. Cells were infected with *P. acnes *at a MOI of 16:1 and immediate close contact between bacteria and cells was achieved by centrifugation of the flask for 10 min at 700 g. Non-infected cells were used as controls.

For the TLR2 blocking experiments, RWPE-1 cells were pre-treated with mouse anti-mouse/human TLR2 mAb (clone T2.5 [13] cat.code: mab-mtrl2, InVivoGen, San Diego, USA) at the concentration 100 ng/ml for 1 h. The cells were then infected with *P. acnes *as described above.

Supernatants were harvested after 24 h and 48 h. Supernatants were cleared from particles by centrifugation 10 min at 12000 g, stored at -20C and later assayed for IL-6, IL-8 and GM-CSF by ELISA (R&D systems, Minneapolis, Minnesota) according to manufacturer's instruction.

### RNA preparation and Reverse Transcription PCR

Cells were seeded at a density of 1 × 10^6 ^in a 25 cm^2 ^culture flask in normal growth medium. After 48 h, cells were washed in PBS and the medium were changed to DMEM without FCS and PEST. Cells were infected with *P. acnes *at a MOI of 16:1 and immediate close contact between bacteria and cells were achieved by centrifugation of the flask for 10 min at 700 g. Total RNA was prepared after 0 h and 24 h using RNeasy Mini kit (Qiagen, Hilden, Germany) with the on-column DNase treatment step according to manufacturer's instruction. Cells were trypsinised using 0,05% (w/v) trypsin/EDTA, lysed in 350 μl RTL buffer and homogenized in a TissueLyser with Stainless steel Beads, 5 mm (Qiagen, Hilden, Germany).

RNA concentration and purity were assessed in a NanoDrop^© ^ND-1000 spectrophotometer (Thermo scientific, Wilmington, USA) at A260 and the ratios of A260:A230 and A260:280.

Complementary DNA (cDNA) was generated from one μg total RNA using RT^2 ^First strand kit (SABiosciences, Frederick, MD, USA) according to the manufacturer's instruction. Quality of the cDNA was verified by PCR array housekeeping genes: beta-2-microglobulin, hypoxanthine phosphoribosyltransferase 1, ribosomal protein L13a, glyceraldehyde-3-phosphate dehydrogenase, beta-actin using primers from (SABiosciences, Frederick, MD, USA).

### Real-time Quantitative PCR

Gene expression analysis measuring transcription of 84 inflammation associated genes was conducted using the RT^2 ^Profiler PCR Array, Human Toll-Like Receptor Signaling Pathway PAHS-018A (SABiosciences, Frederick, MD, USA) according to manufacturer's instruction. Real-time PCR detection was performed with an IQ™5 instrument (Bio-Rad, Hercules, CA, USA).

Complete list of genes analyzed by the array can be found at: http://www.SABiosciences.com

### Data Analysis

Relative gene expression was calculated with the ΔΔC_t _method in the web-based software package for RT^2 ^Profiler PCR array systems (SABiosciences, Frederick, MD, USA).

### Statistical Methods

Due to the small sample size (*n *= 3), a permutation test was used to test possible regulation [38]. A null hypothesis corresponding to no regulation was tested for each gene and each protein concentration and rejected for *p *= 0.05.

## Authors' contributions

JBD carried out the tissue culture infections, the mRNA assays and the protein quantification. OA participated in the experimental design. PB performed the statistical analysis. FE initiated the study and participated in its design. JO participated in the design of the study, performed pilot studies of experimental conditions and drafted the manuscript. All authors read and approved the final manuscript.
